# Dermal fibroblasts in Hutchinson-Gilford progeria syndrome with the lamin A G608G mutation have dysmorphic nuclei and are hypersensitive to heat stress

**DOI:** 10.1186/1471-2121-6-27

**Published:** 2005-06-27

**Authors:** Mauro Paradisi, Dayle McClintock, Revekka L Boguslavsky, Christina Pedicelli, Howard J Worman, Karima Djabali

**Affiliations:** 1 VII Divisione, Dermatologia Pediatrica, Istituto Dermopatico Dell'Immacolata IRCCS, Rome, Italy; 2 Department of Dermatology, Columbia University, College of Physicians & Surgeons, New York, New York, USA; 3 Department of Medicine and Department of Anatomy and Cell Biology, Columbia University, College of Physicians & Surgeons, New York, New York, USA

## Abstract

**Background:**

Hutchinson-Gilford progeria syndrome (HGPS, OMIM 176670) is a rare sporadic disorder with an incidence of approximately 1 per 8 million live births. The phenotypic appearance consists of short stature, sculptured nose, alopecia, prominent scalp veins, small face, loss of subcutaneous fat, faint mid-facial cyanosis, and dystrophic nails. HGPS is caused by mutations in *LMNA*, the gene that encodes nuclear lamins A and C. The most common mutation in subjects with HGPS is a *de novo *single-base pair substitution, G608G (GGC>GGT), within exon 11 of *LMNA*. This creates an abnormal splice donor site, leading to expression of a truncated protein.

**Results:**

We studied a new case of a 5 year-old girl with HGPS and found a heterozygous point mutation, G608G, in *LMNA*. Complementary DNA sequencing of RNA showed that this mutation resulted in the deletion of 50 amino acids in the carboxyl-terminal tail domain of prelamin A. We characterized a primary dermal fibroblast cell line derived from the subject's skin. These cells expressed the mutant protein and exhibited a normal growth rate at early passage in primary culture but showed alterations in nuclear morphology. Expression levels and overall distributions of nuclear lamins and emerin, an integral protein of the inner nuclear membrane, were not dramatically altered. Ultrastructural analysis of the nuclear envelope using electron microscopy showed that chromatin is in close association to the nuclear lamina, even in areas with abnormal nuclear envelope morphology. The fibroblasts were hypersensitive to heat shock, and demonstrated a delayed response to heat stress.

**Conclusion:**

Dermal fibroblasts from a subject with HGPS expressing a mutant truncated lamin A have dysmorphic nuclei, hypersensitivity to heat shock, and delayed response to heat stress. This suggests that the mutant protein, even when expressed at low levels, causes defective cell stability, which may be responsible for phenotypic abnormalities in the disease.

## Background

Hutchinson-Gilford progeria syndrome (HGPS, OMIM 176670) is a rare sporadic disorder with an incidence of 1 per 8 million live births. Birth weight and appearance are usually normal, but growth typically becomes retarded at the age of 1 year. Phenotypic features include short stature, sculptured nose, alopecia, prominent scalp veins, small face, subcutaneous fat loss, faint mid-facial cyanosis, and dystrophic nails. Features occurring in the skin during late adulthood of normal individuals, such as hair greying, hair loss, and skin thinning, occur in the first few years of life in subjects with HGPS [1-3]. Most subjects die in their teenage years from cardiac complications of coronary artery disease or stroke due to widespread arteriosclerosis [2].

The diagnosis of HGPS was formerly based on the criteria of growth retardation and prematurely aged phenotype in children. In 2003, however, mutations in the *LMNA *gene that encodes nuclear lamins A and C were identified as responsible for this syndrome [4-6]. As such, HGPS belongs to the group of diseases caused by mutations in *LMNA*, sometimes referred to as "laminopathies," which also includes disorders of striated muscle, peripheral nerve and partial lipodystrophy syndromes [7,8]. The *LMNA *mutation present in the majority of subjects with HGPS is a *de novo *heterozygous base change (GGC>GGT) within exon 11 of the *LMNA *gene, which does not cause an amino acid substitution (G608G) but creates an abnormal splice donor site [4,5].

Nuclear lamins are members of the intermediate filament protein superfamily [9,10]. They are the building blocks of the nuclear lamina, a fibrous proteinaceous meshwork underlying the inner nuclear membrane [11]. Nuclear lamins have an extra 42 amino acids (six heptads) in coil 1B compared to cytoplasmic intermediate filament proteins [12,13]. Nuclear lamins also contain two unique sequences: a nuclear localization signal in the tail domain [14], and, except for lamin C, a carboxyl-terminal CAAX box (cysteine-aliphatic-aliphatic-any amino acid), a target for isoprenylation [15-18]. In the human genome, 3 distinct loci encode lamins. *LMNA *is located on chromosome 1q21.2 [19,20] and encodes 4 lamins by alternative RNA splicing: A, C, AΔ10, and C2 [21]. Lamin A is synthesized as a precursor, prelamin A, from which 17 amino acids are removed from the carboxyl-terminal by endoproteolysis after isoprenylation. There are 2 B-type lamin genes: *LMNB1 *on chromosome 5q23-q31.1 that encodes lamin B1 [20,22], and *LMNB2 *on chromosome 19p13.3 [23] that encodes lamin B2 [24] and lamin B3, an alternatively spliced isoform expressed in germ cells [25].

We recently identified a new female subject with HGPS from Italy. We now show that she has the most common heterozygous point mutation, G608G, in *LMNA *resulting in expression of the prelamin A mutant with 50 amino acids deleted from the carboxyl-terminal tail domain. We analyzed the nuclear morphology and growth characteristics of these fibroblasts, and for the first time demonstrate that cells from a subject with HGPS exhibit increased susceptibility to heat stress.

## Results

### Clinical description of a new subject with HGPS

**Figure 1:**
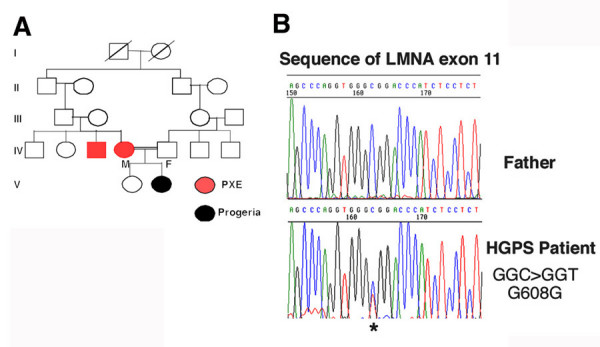
**Characterization of a new subject with HGPS. **(A) Pedigree of the subject with HGPS. Filled black circle indicates the proband; red circle and square respectively represent the female (mother of proband) and male (uncle of proband) with pseudoxanthoma elasticum (PXE). A diagonal line indicates a deceased individual. Double lines indicate consanguinity. (B) Short portion of the *LMNA *sequence within exon 11. The paternal sequence (Father) corresponds to the wild type *LMNA *sequence. The affected subject (HGPS Patient) has a heterozygous C to T transition (indicated by a star) at nucleotide 1824 in exon 11 of the *LMNA *sequence.

The female subject is the second child from consanguineous parents (second cousins) (Fig. 1A). The subject's mother and an uncle (Fig. 1A, red) were affected with pseudoxanthoma elasticum, an inherited disorder of connective tissue. At birth, the subject had cutaneous xerosis and mild skin indurations of the lower limbs. At 1 month, she developed more severe skin stiffening on the lower limbs, trunk, and extensor areas on the forearms, accompanied by functional limitation in leg extension. Mild perioral cyanosis was also visible. A skin biopsy was performed at 2 years of age and histological analysis revealed fibrous thickening of the lower dermis, subcutaneous septa, and fascia, accompanied by a few mucinous deposits. Weigert staining showed pronounced rarefactions of elastic fibers. A dermal fibroblast culture was established from the biopsy sample. Physical examination of the subject at age 5 years revealed loss of subcutaneous tissue, especially on the face and limbs, and thickening of the skin that appeared shiny and taut in most areas. She also had a small face with a recessed chin, thin beaked nose, small ears, prominent eyes, prominent scalp veins, and alopecia.

### Detection of a *LMNA *mutation encoding a truncated prelamin A

We sequenced all 12 exons of *LMNA *that comprise the lamin A/C coding region and splice junctions of the subject's DNA using previously described oligonucleotide primers [26]. We found a heterozygous C to T transition at nucleotide 1824 in exon 11 of *LMNA*, which created a silent point mutation at codon 608 (GGC>GGT; G608G) (Fig. 1B). We also sequenced exon 11 from the father (Fig. 1B) and the mother (data not shown) and found no mutation in either.

**Figure 2:**
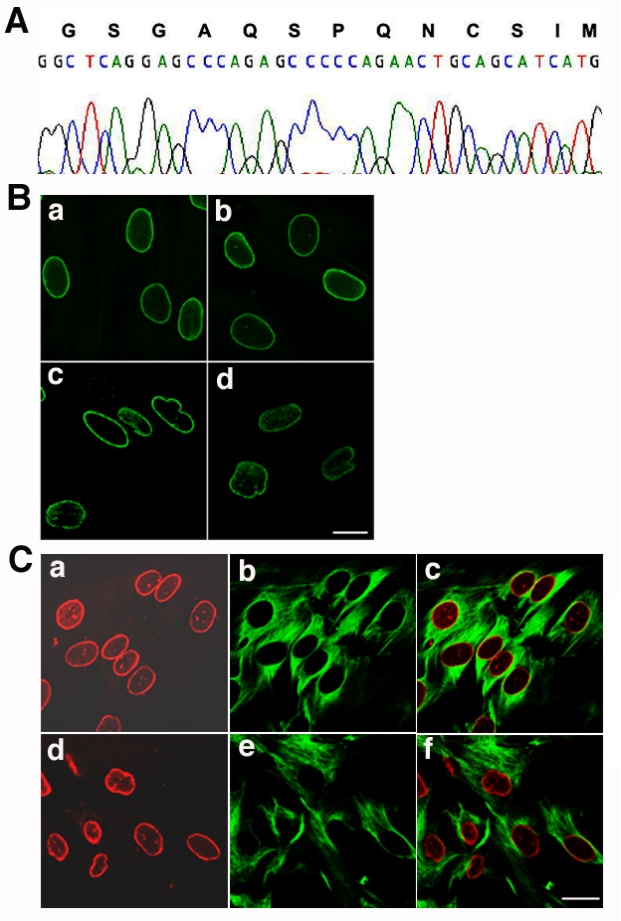
**Sequencing of the mutant prelamin A cDNA and fibroblast morphology in the subject with HGPS. **(A) Sequence of cDNA encoding mutant prelamin A in the subject with HGPS. Mutant prelamin A cDNA sequence from nucleotide 1804 to the end of the coding region and the corresponding deduced amino acid sequence are shown. (**B**) Confocal microscopic analysis of dermal fibroblast in primary culture from a control (a and b) and the subject with HGPS (c and d). Labelling was performed with anti-lamin A/C antibodies. Note the presence of irregularly shaped nuclear envelopes in many of the subject's fibroblasts. (**C**) Double label immunofluorescence microscopic analysis of fibroblasts from an unaffected control (a to c) and subject with HGPS (d to f) labelled with anti-lamin B1 (red)) and anti-vimentin (green) antibodies. Bars, 10 μm.

Amplification of cDNA fragments by RT-PCR corresponding to nucleotides 1561 to 2010 of prelamin A mRNA showed that the wild type fragment was 449 nucleotides and the mutant fragment 299 nucleotides. The mutant sequence was 150 nucleotides shorter than the wild type, corresponding to a deletion of 50 amino acids. Figure 2A shows the sequence of the mutant lamin A cDNA from nucleotide 1804 (in the codon for amino acid 602) to the last amino acid codon. The deducted amino acid sequence of the mutant from residue 602 to the carboxyl-terminus is GSGAQSPQNCSIM (Fig. 2A).

### Nuclear envelope morphology in HGPS fibroblasts

We examined the nuclear envelope morphology of dermal fibroblasts from control individuals and from the subject with HGPS after 10 or less passages in primary cultures. Cells were fixed and immunostained with anti-lamin A/C antibodies and examined by confocal microscopy. Nuclei of control fibroblasts were mostly regular in size and shape, and appeared generally round or ovoid (Fig. 2B, a and b). In contrast, the size of nuclei in the subject's fibroblasts was more variable, and the nuclear envelopes had convex "blebs" or herniations projecting towards the cytoplasm (Fig. 2B, c and d). By direct count of 1,000 cells in 3 different cultures from the subject and controls, we determined that 19% of nuclei in the subject's fibroblasts had irregularities in nuclear envelope shape compared to only 4% of control cells, in which the dysmorphic nuclei exhibited less severe abnormalities. Despite abnormalities in nuclear envelope size and shape in fibroblasts from the subject with HGPS, there were no gross abnormalities in the localization of lamins A/C (Fig. 2B, c and d) or lamin B1 (Fig. 2C, d). Furthermore, the vimentin network appeared normal (Fig. 2C, e). Staining with 4',6-diamidino-2-phenylindole (DAPI) did not reveal obvious defects in chromatin density (data not shown).

**Figure 3:**
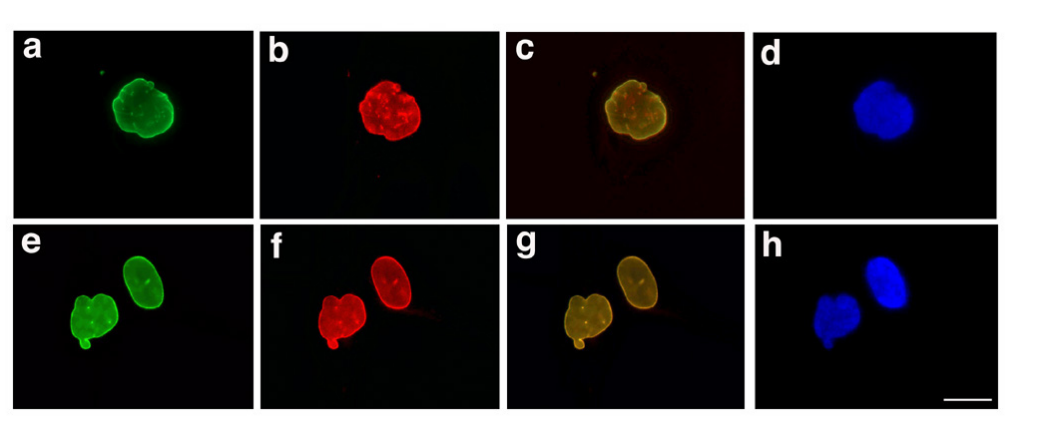
**Comparison of lamin A/C localization with lamin B1, emerin and chromatin in dysmorphic nuclei of fibroblasts from the subject with HGPS. **Upper panel shows micrographs of fibroblasts from the subject with HGPS labelled with anti-lamin A/C antibodies (a), anti-lamin B1 antibodies (b), the merged signal (c) and DAPI (d). Lower panel shows micrographs of fibroblasts from the same subject labelled with anti-lamin A/C antibodies (e), anti-emerin antibodies (f), the merged signal (g), and DAPI (h). Bar, 10 μm.

We further examined the localizations of A-type lamins, B-type lamins, and emerin, an integral protein of the inner nuclear membrane, using double labelling immunofluorescence microscopy. We examined the most abnormal appearing nuclei in the subject's cells, carefully analyzing the localization of lamin B1 (Fig. 3b) and emerin (Fig. 3f) compared to A-type lamins (Fig. 3a and 3e). At the nuclear periphery of the subject's most dysmorphic nuclei, labelling of A-type and B-type lamins is super-imposable (Fig. 3c). Even in sites where the nuclear envelope contained "blebs," both A-type and B-type lamins were colocalized. Emerin also colocalized with A-type lamins in the same distribution pattern (Fig. 3g). When intranuclear lamin A/C labelling was present, emerin was found in the same structures, suggesting that they represented invaginations of the nuclear envelope. Concurrent DAPI staining did not demonstrate a gross detachment between the nuclear lamina (A-type and B-type lamin labelling) and chromatin, even in the most abnormally shaped nuclei (Fig. 3 a to d). Lamin A/C, B1 and emerin labelling were in close apposition to the DNA labelling, suggesting that the nuclear envelope and lamina remained in close approximation to chromatin. DNA labelling detected in the herniated areas of nuclei also was associated with labelling by anti-lamin A/C, anti-lamin B1 and anti-emerin antibodies (Fig. 3d and 3h).

**Figure 4:**
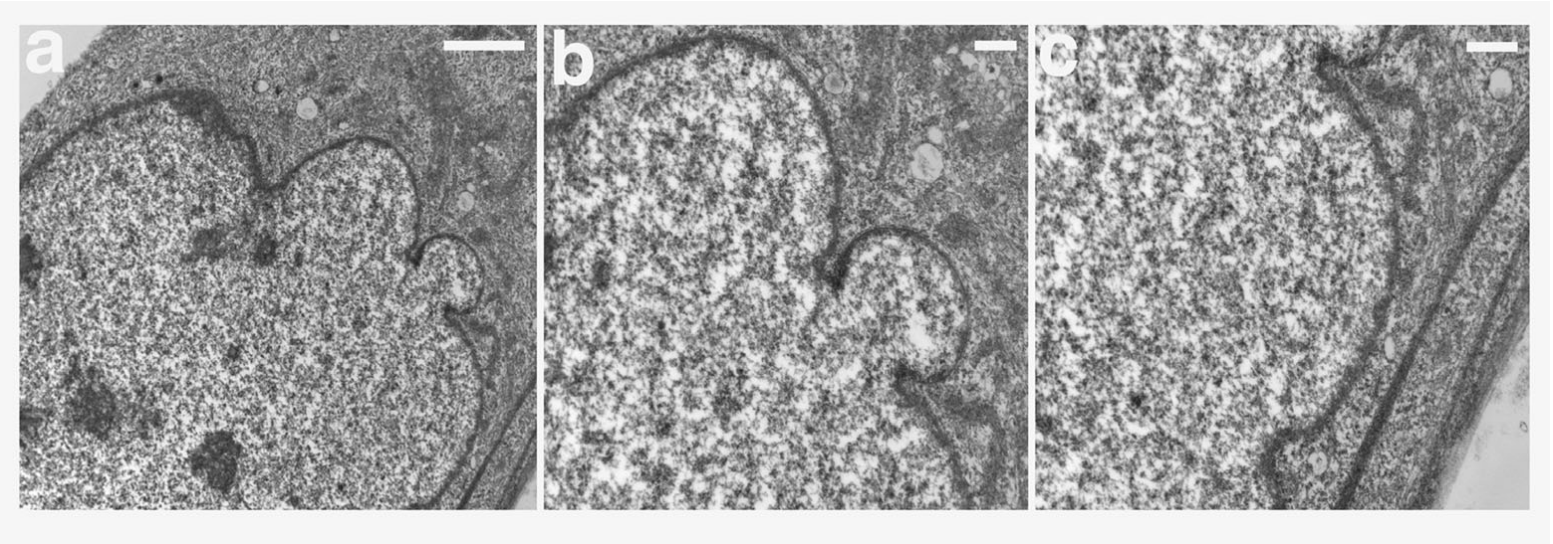
**Ultrastructural analysis of the nuclear envelope in fibroblasts from the subject with HGPS. **Low magnification transmission electron microscopic image of a passage 10 PT001 nucleus showed several herniations (a). Two higher-magnification images of the same nucleus at sites of blebs (b and c) showed a close apposition of the chromatin to the nuclear envelope. In a, b, and c the nucleus is to the left. Scale bars correspond to 2 μm in panel a, and 500 nm in panels b and c.

We carried out an ultrastructural analysis of cultured fibroblasts from the subject with HGPS using transmission electron microscopy. The electron micrographs clearly showed that chromatin remained in contact with the nuclear envelope (Fig. 4a to 4c). Analysis of a dysmorphic nucleus with 3 blebs (Fig. 4a) revealed that the chromatin also remained attached to the nuclear envelope at those sites (Fig. 4b and 4c).

### Analysis of lamin expression by Western blotting

**Figure 5:**
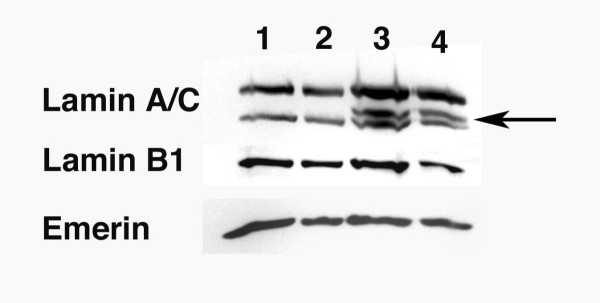
**Detection of lamins A and C, lamin B1 and emerin by Western blotting in fibroblasts from the subject with HGPS. **Fibroblasts from unaffected controls (lanes 1 and 2) and the subject with HGPS (lanes 3 and 4) were lysed in Laemmli buffer, and whole-cell extracts corresponding to 0.5 × 10^6 ^cells (lanes 1 and 3) and 0.25 × 10^6 ^(lanes 2 and 4) were analyzed by immunoblotting using anti-lamin A/C (lane: Lamin A/C), anti-lamin B1 (lane: Lamin B1) and anti-emerin (lane: Emerin) antibodies. Arrowhead points to the position of the prelamin A mutant in lanes 3 and 4.

We performed Western blot analysis on total protein extractions from cultured fibroblasts at passage number 10. Anti-lamin A/C antibodies that recognize a sequence predicted to be within the truncated G608G prelamin A mutant, in addition to lamin A and C bands, detected the mutant protein in the subject's cells migrating above the band corresponding to lamin C (Fig. 5, panel Lamin A/C, lanes 3 and 4, arrow). This protein was not detected in extracts of control fibroblasts (Fig. 5, panel Lamin A/C, lanes 1 and 2). In Western blots containing equal amounts of cell extracts, the lamin A and lamin C signals were approximately the same for subject and control cells (Fig. 5; panel Lamin A/C). This was confirmed by densitometric analysis of autoradiograms of 4 separate Western blots from subject and control cells. In addition, the signals on Western blots obtained using anti-lamin B1 and anti-emerin antibodies showed no obvious differences between the subject and unaffected individuals (Fig. 5, panels Lamin B1 and Emerin). Hence, at low passage number in primary culture, fibroblasts from a subject with HGPS express a mutant truncated prelamin A, but lamin A, lamin C, lamin B1, and emerin are expressed at approximately normal levels.

### Growth rate of fibroblasts from a subject with HGPS in early passage primary culture

We examined the growth rate of cultured primary dermal fibroblasts from the subject with HGPS. At each cellular passage, the total number of cells from control and proband was assessed by direct count prior to plating. The mean number of harvested HGPS fibroblasts was comparable to the number obtained with two control fibroblast lines derived from unaffected individuals at passage numbers in culture below passage 10. Dermal fibroblasts from the subject with HGPS therefore had a similar growth rate to the control counterparts and did not exhibit gross cell cycle defects at low passage numbers in culture.

### Dermal fibroblasts from a subject with HGPS are hypersensitive to heat stress

**Figure 6:**
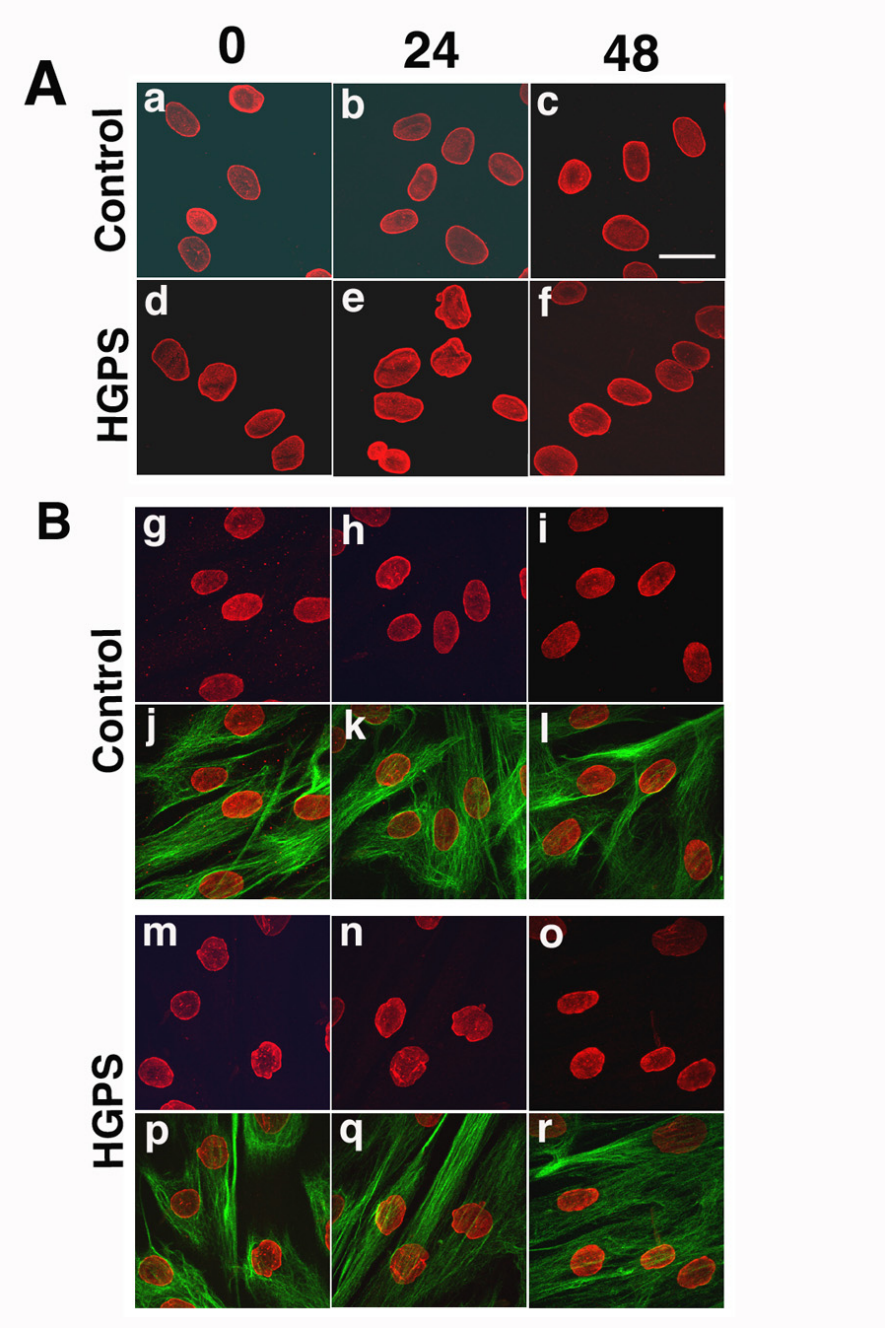
**Confocal analysis of dermal fibroblasts after heat shock stress. **Cells were incubated at 45°C for 30 minutes, then either immediately fixed (time 0) or allowed to recover for 24 or 48 hours at 37°C. Cells were processed for indirect immunofluorescence labelling using anti-lamin A/C antibodies (panel A, a to f), anti-lamin B1 antibodies (panel B, g to r) and anti-vimentin antibodies (panel B j to i and p to r). Control and HGPS fibroblasts are indicated. (**A**) Fibroblasts were immunostained with anti-lamin A/C antibodies. Note the increased number of dysmorphic nuclei in HGPS fibroblasts compared to control 24 hours after recovery from heat shock. (**B**) Fibroblasts were immunostained with anti-lamin B1 and vimentin at times indicated after heat shock. Bar, 10 μm.

To evaluate the resistance to stress of the nuclear envelope, fibroblasts from the subject with HGPS and control individuals were heat shocked for 30 minutes at 45°C. Cells were then either fixed immediately (time 0) or incubated at 37°C for 24 and 48 hours to allow for recovery. Nuclear shape was examined by microscopy after labelling with anti-lamin A/C or anti-lamin B1 antibodies at time 0, 24 hours and 48 hours after heat shock (Fig. 6). In control cells, nuclear shape and distribution of A-type and B-type lamins were not altered by heat shock (Fig. 6A a to c and Fig. 6B g to i, respectively). In contrast, extensive nuclear deformations appeared in the cells from the subject with HGPS (Fig. 6A d to f and Fig. 6B m to o). The subject's fibroblasts showed an increase in the number of cells with altered nuclei immediately after heat shock. Nuclear envelopes were deformed, many had a ruffled appearance, and some had "pleats" or "folds" (Fig. 6, d and m, time 0). The number of irregular nuclei increased to nearly 70 % after 24 hours, as determined by direct count of 1,000 nuclei in cells on different coverslips. Some nuclei were more severely affected than others and showed "blebbing" or nuclear lobe formation 24 hours after heat shock (Fig. 6, e and n, time 24). This type of nuclear damage was never observed in the control cells (Fig. 6; a, b, g, and h). At 48 hours after heat shock, nuclei with invaginations were no longer observed in the fibroblasts from the subject with HGPS (Fig. 6; f and o), suggesting that the cells with severely dysmorphic nuclei died and detached from the coverslips during processing for immunofluorescence microscopy. Fibroblasts remaining on the coverslips appeared to have recovered from the stress, as their nuclei showed a less ruffled appearance and the irregularities resembled those observed prior to the heat shock (Fig. 6; f and o, time 48). There was no apparent rearrangement of the vimentin cytoplasmic intermediate filament network after heat shock and 24 and 48 hours after recovery in the subject's and control fibroblasts (Fig. 6; p to r).

Survival rates of fibroblasts subjected to heat shock were evaluated and compared to unheated cells (Table 1). At 24 hours after recovery, 25% of the subject's cells were lost compared to the number of cells prior to treatment. In contrast, the total number of control fibroblasts increased by 39%. This suggests that growth of control cells was not significantly affected by heat shock, as they continued to divide. After 48 hours, the subject's fibroblasts appeared to have recovered from stress, as the number of cells increased, reaching approximately the same number of cells per dish as before heat shock. From 24 hours to 48 hours after heat shock, the number of cells from the subject with HGPS increased by 30%. Control cells continued to increase by an average of 36% at 48 hours after heat shock.

**Table 1:** Percentage of cells recovered after heat shock treatment. Values shown are mean percentages (plus or minus standard errors, n = 3) of fibroblasts collected from 10 cm culture dishes prior to and 24 hours and 48 hours after heat shock for a period of 30 minutes at 45°C.

	**% of fibroblasts recovered after heat treatment**			
	Before heat shock	Hours after heat shock		

		0	24	48
Control	100	97.6 ± 0.9	139.2 ± 1.5	175 ± 2.9
HGPS	100	95.8 ± 1.5	74.6 ± 5.1	104.9 ± 5.8

These experiments indicate that control cells recovered rapidly from heat shock, since no changes were apparent in their growth rate 24 and 48 hours later. However, fibroblasts from the subject with HGPS were hypersensitive to heat shock, and the number with dysmorphic nuclei was high after treatment. Their recovery was only observed 48 hours after heat shock, when cell numbers started to increase, suggesting that surviving cells had begun to divide again.

## Discussion

We report another subject with HGPS who has the *LMNA *G608G mutation. So far, 21 out of 25 reported cases of HGPS genetically analyzed have this point mutation [4,5,27], making it a molecular signature for HGPS. Sequencing of parental DNA, when available, has shown that none of them carried this mutation, indicating that it is a *de novo *mutation. These findings also show that HGPS is an autosomal dominant disease possibly resulting from germinal mosaicism [4].

The *LMNA *G608G mutation creates an abnormal splice donor site producing mRNA with 150 nucleotides deleted from the prelamin A coding sequence. The encoded truncated protein is predicted to be missing residues 607 to 656 of prelamin A and to retain the CAAX box for the prenylation at its carboxyl-terminus. As the "upstream" endoproteolytic cleavage site of wild type prelamin A is deleted [15], the truncated prelamin A in HGPS may remain prenylated; however, this remains to be shown experimentally.

To understand the cellular basis of HGPS, we analyzed the morphology dermal fibroblasts from our subject, which express the mutant protein. We studied the distribution of A-type lamins in primary fibroblast cultures at early cellular passage numbers (less than or equal to 10) to better reflect the *in vivo *distribution of proteins. We observed nuclear abnormalities in a subpopulation of HGPS fibroblasts, where dysmorphic nuclei had "blebs" surrounded by A-type lamins. We did not observe defects in the lamin A/C meshwork in "blebs" of the most dysmorphic nuclei, and chromatin distribution appears normal even within the 'blebs." The fibroblasts did not have gross anomalies of the localization of lamin B1 or emerin, even when the lamina network appeared ruffled.

Only a subpopulation of cultured fibroblasts from our subject with HGPS had dysmorphic nuclei. Furthermore, the nuclear abnormalities observed in these fibroblasts may have been less dramatic than those reported in cell lines from other individuals with HGPS [28]. Nuclear abnormalities in fibroblasts from our subject with HGPS also differed from those reported in fibroblasts from subjects with Dunnigan-type familial partial lipodystrophy, a condition caused by different autosomal dominant *LMNA *mutations. Cells from these subjects have defects in lamin A/C distribution in nuclear envelope "blebs," and there is also a loss of lamin B1 staining around the nuclear envelope "blebs" and sometimes at the nuclear pores [29]. Despite these subtle differences between cells from subjects with HGPS and subjects with other laminopathies, generally similar morphological alterations invariably occur in some portion of cultured fibroblasts from subjects with different lamin A/C mutations [21,29,30]. Grossly similar alterations are also observed in fibroblasts from *Lmna *knockout mice [31,32]. Based on these nuclear envelope abnormalities common to all laminopathies, it appears that A-type lamins play a crucial role in the maintenance of the size and shape of the nucleus. These alterations in nuclear morphology could potentially lead to abnormalities in cell growth or structural stability.

We did not observe differences in the proliferation of fibroblasts from our subject with HGPS compared to fibroblasts from unaffected controls at passage numbers below 10 in primary culture. A recent report suggested that, after a certain number of passages, fibroblasts from subjects with HGPS were no longer able to proliferate at a similar rate as fibroblasts from unaffected controls [28]. Growth inhibition was recently reported for other cell lines from subjects with HGPS, where after a certain number of doublings, the cells rapidly entered a senescence phase [33,34]. Fibroblasts from *Lmna *knockout mice are prone to apoptosis when derived from newborns, while fibroblasts derived from knockout embryos can grow in culture for a restricted number of passages [30].

With regards to cell stability, we observed that fibroblasts from our subject with HGPS cells were more susceptible to damage from heat shock than controls. The subject's cells in culture had an increased number of dysmorphic nuclei and enhanced cell death within the first 24 hours after the heat shock. Fibroblasts from the subject with HGPS appeared to be able to eventually recover from heat stress, since they started to grow again after a delay of 24 hours. These findings suggest that fibroblasts form subjects with HGPS are fragile and more readily damaged or killed after stress. Increased nuclear fragility to mechanical stress and head shock has also been reported in fibroblasts from mice lacking lamins A and C and human subjects with Dunnigan-type familial partial lipodystrophy [29,35].

## Conclusion

Cells from a subject with HGPS and the lamin A G608G mutation have dysmorphic nuclei and an increased susceptibility to damage by heat shock. Our findings suggest that these cells may also be sensitive to other types of stress, such as metabolic or mechanical. Improving the cellular response to stress may be one possible way of helping individuals with HGPS and other laminopathies.

## Methods

### Clinical material

After obtaining informed consent, we obtained blood from a subject with a clinical diagnosis of HGPS, her farther, and her mother. Genomic DNA was isolated from peripheral blood according to standard techniques [36]. A skin biopsy had been performed from the right leg when the subject was 2 years of age for histological examination, and a dermal fibroblast culture was concomitantly established from the same tissue sample. Two control dermal fibroblast cultures were established from neonatal foreskin and skin of a 9 year-old who underwent surgery without any known diseases. Primary dermal fibroblasts were maintained in DMEM containing 15 % fetal calf serum, 2 mM glutamine, 100 IU/ml penicillin, and 100 mg/ml streptomycin for a maximum of 12 passages. The Columbia University Medical Center Institutional Review Board approved the use of cells from human subjects.

### *LMNA *sequencing

All exons of the lamin A/C coding region of *LMNA *and splice junctions were amplified by PCR from genomic DNA using primers described previously [26]. Amplified DNA was sequenced directly using an ABI Prism 310 Automated Sequencer and the ABI Prism Big Dye Terminator Cycle Sequencing Ready Reaction Kit (PE Applied Biosystems) following purification in a Centriflex Gel Filtration Cartridges (Edge Biosystems). Mutations were identified by visual inspection and comparison with sequences generated from unrelated, unaffected individuals, and the GeneBank reference sequence.

### Prelamin A cDNA sequencing

Total RNA was extracted from cultured dermal fibroblasts using Trizol reagent according to the manufacturer (Invitrogen). Using the forward primer (5' GGCTGCGGGAACAGC 3') and the reverse primer (5' CTGGCAGGTCCC 3'), we amplified by RT-PCR the cDNA spanning nucleotides 1561 to 2010 of the prelamin A coding sequence. Amplified DNA products were purified and subcloned into the pGBT9 (Invitrogen). Clones were sequenced using the forward and reverse primers described above. The mutant sequence was verified by sequencing 3 independent clones generated from 3 different RT-PCRs.

### Indirect immunofluorescence microscopy

Primary cultures of dermal fibroblasts were grown on glass coverslips, washed with PBS, fixed in methanol at -20°C, and then processed for indirect immunofluorescence as described previously [37,38]. Rabbit antibodies directed against A-type and B-type lamins were kindly provided by Dr. N. Chaudhary and have been previously described [39,40]. Secondary antibodies were affinity purified Alexa Fluor 488 goat anti-rabbit or anti-mouse IgG antibodies (Molecular probes) and Cy3-conjugated goat anti-rabbit or anti-mouse (Jackson ImmunoResearch Laboratories). For double immunofluorescence microscopy with the rabbit anti-A-type lamin antibodies, we used a mouse monoclonal anti-emerin clone 4G5 (Novacastra Laboratories) or a previously described human anti-lamin B antiserum [41], which was kindly provided by Dr. J.-C. Courvalin. Cells were also examined with anti-vimentin antibodies [42], kindly provided by Dr. S. D. Georgatos, and appropriate secondary antibodies. All samples were also counterstained with DAPI (Sigma-Aldrich) and slides were examined using a confocal microscope.

### Transmission Electron Microscopy

Cells were fixed with 2.5% glutaraldehyde in 0.1 M Sorenson's buffer (PH 7.2) for an hour, and postfixed with 1% OsO4 in Sorenson's buffer for one hour. Enblock staining was performed using 1% tannic acid. After dehydration cells were embedded in a mixture of Lx-112 (Ladd Research Industries, Inc.) and Embed-812 (EMS, Fortwashington, PA). Thin sections were cut on a MT-7000 ultramicrotome. Sections were stained with uranyl acetate and lead citrate, and examined under a JEOL JEM-1200 EXII electron microscope.

### Western blot analysis

Total cellular protein extracts were isolated from dermal fibroblasts in primary cultures at passage number 10. Total cell number was determined for each culture, and cell pellets were extracted directly in Laemmli sample buffer [43]. Cells were lysed and equal amounts of extracts (corresponding to the same number of cells) were loaded in parallel on a 6% polyacrylamide gel. After separation by electrophoresis, proteins were transferred to nitrocellulose membranes and incubated with blocking buffer for several hours as described previously [44]. Membranes were incubated with primary antibodies, washed, and then incubated with the corresponding secondary antibody coupled to horseradish peroxidase (Jackson ImmunoResearch Laboratories). Proteins were visualized using the enhanced chemiluminescence's detection system (Amersham Pharmacia Biotech). Signals obtained on the autoradiograms were analyzed by densitometry using Quantity One 1-D analysis software (BioRad Laboratories) on the scanned images.

### Heat shock treatment

Cells were grown in 10 cm diameter dishes in complete medium and transferred from 37°C to 45°C for 30 minutes. Viability was determined prior to heat shock, and at time 0 (after 30 minutes stress), 24 hours, and 48 hours after recovery at 37°C. Attached cells were trypsinized at each time point, collected and counted. For morphological analysis, fibroblasts were grown on coverslips and treated as above. Unheated and heated samples were fixed in methanol at -20°C, and processed for indirect immunofluorescence as described above.

## Authors' contributions

MP identified the new subject with HGPS and provided the clinical data and materials. CP established the HGPS dermal fibroblast primary culture. DM performed the heat shock experiments, and immunohistochemistry experiments. RLB participated in the subcloning and sequencing of the mutant lamin A cDNA. HJW was involved in the subcloning, sequencing of the mutant lamin A cDNA and in manuscript preparation. KD performed the screening of the *LMNA *gene and was responsible for designing and carrying out this work and for preparing the manuscript.
